# Utility of State-Based Basic Screening Survey Reports for National Oral Health Surveillance in Older Adults

**DOI:** 10.5888/pcd18.200471

**Published:** 2021-04-08

**Authors:** Molly Linabarger, Susan O. Griffin, Erin K. Hamilton

**Affiliations:** 1 Deloitte Consulting LLP, Atlanta, Georgia; 2 Division of Oral Health, Centers for Disease Control and Prevention, Atlanta, Georgia; 3 CyberData Technologies, Atlanta, Georgia

## Abstract

**Introduction:**

Untreated dental disease and resulting tooth loss can diminish quality of life for older adults by limiting food choices and discouraging social interaction. Before the Basic Screening Survey (BSS) for older adults, no clinical data were available to monitor the oral health of older adults in long-term care (LTC) facilities at the national level or older adults overall at the state level. Although BSS is widely used, no guidelines exist to ensure the validity, reliability, and comparability of survey information across states. We examined BSS content to help establish reporting guidelines and synthesized findings across states for older adults living in LTC.

**Methods:**

We systematically reviewed BSS reports published from 2011–2019, assessing how oral health outcomes were measured and reported. For reports that included statewide estimates for LTC residents, we calculated the mean, median, and ranges of 3 preventable oral health conditions and 4 indicators of tooth loss.

**Results:**

We found wide variation in reporting of sampling, screening, and statistical methods, as well as in indicators of tooth loss. Median prevalence of untreated tooth decay and edentulism (total tooth loss) among LTC adults in 11 states was almost twice that for community-dwelling adults in a national survey.

**Conclusions:**

The substantial variation in BSS reporting highlights the potential benefits of adopting standardized guidance, which could improve the utility of BSS. Poor oral health outcomes among LTC residents underscore the importance of systematic monitoring of the oral health of this vulnerable population.

**Summary**

**What is already known on this topic?**

The Basic Screening Survey (BSS) fills a key surveillance gap in monitoring oral health among older adults living in long-term care (LTC). However, guidance is limited on how to report study methods and specifications for outcome measurements, which would enable comparison of BSS findings across states.

**What is added by this report?**

This review of BSS reports found wide variation in reporting of study methods. Median prevalence of untreated caries and edentulism for LTC adults across 11 states was approximately twice those from national surveys of community-dwelling adults.

**What are the implications for public health practice?**

Reporting guidelines and more detailed instruction can improve the comparability and usefulness of resource-intensive BSS surveys.

## Introduction

Although largely preventable, untreated dental diseases and resulting tooth loss are prevalent among community-dwelling older adults (1). These conditions can diminish their quality of life by limiting food choices and discouraging social interactions (2). National data indicate significant disparities in tooth decay and tooth loss among community-dwelling older US adults by income, race/ethnicity, and educational attainment (1,3). Data also indicate a continuing decline in complete tooth loss since the 1960s. Although this trend suggests improved quality of life, it could also lead to more caries and periodontal disease if older adults do not have access to effective preventive dental services (3).

For older adults living in long-term care (LTC) facilities (6.5% of all older adults [4]), no national data on clinical oral health status are available. The National Health and Nutrition Examination Survey, a survey with a combination of interviews and physical examinations and the only national survey with a clinical assessment of oral health, does not sample institutionalized people. Thus, a critical need exists for timely information to monitor the oral health status of older adults living in LTC facilities.

To have oral health data for older adults, the Association of State and Territorial Dental Directors (ASTDD) developed the older adult Basic Screening Survey (BSS) in the early 2000s (5). As of 2020, at least 26 states have reported conducting BSS among older adults since the development of the survey. Although a stated objective of BSS is to generate estimates of oral health status that are comparable across states, our preliminary review of BSS reports suggests that what is reported varies across states. Currently, no established reporting checklist (similar to GATHER [Guidelines for Accurate and Transparent Health Estimates Reporting] for epidemiologic and CHEERS [Consolidated Health Economic Evaluation Reporting Standards] for economic studies) exists for a BSS report (6,7).

Standardized reporting would enable users to assess the quality of data in a BSS report and also improve comparability across states. Thus, a review of heterogeneity in reporting across states could provide foundational information for developing a BSS checklist.

The aims of this study were to systematically review publicly available state BSS reports to 1) assess heterogeneity in the reporting of how BSS was conducted and the findings across states and 2) synthesize oral health–related estimates for older adults living in LTC facilities.

## Methods

In 2019 and 2020, we systematically reviewed available published BSS reports to accomplish the 2 study aims.

### Items used in assessing reporting of heterogeneity

The BSS protocol provides guidance on how to conduct a BSS among community-dwelling older adults and older adults living in LTC facilities, including survey methods and a list of recommended and optional oral health indicators to be collected (5) (Appendix). Complete information on the protocol is available to ASTDD members.

Although ASTDD highlights the importance of training and calibrating examiners and using sound statistical methods and reporting, it does not prescribe specific methods, acknowledging that some members may have limited resources, which could have greater impact than statistical rigor. To assess heterogeneity in how BSS is conducted, we examined whether the following items were included in each BSS report: 1) screener calibration (ie, training, practice, and discussion for consistent results both within and between screeners); 2) inclusion of a consultant; 3) sampling methods; 4) sample size considerations; 5) survey response rate; 6) adjustment of data to make findings more representative; and 7) precision and error measures.

To assess heterogeneity in the reporting of oral health status, we focused on preventable outcomes (ie, untreated dental caries, periodontal disease, and urgent treatment need) and outcomes known to affect a person’s ability to eat healthy foods (ie, dentate status, mean number of missing teeth, having upper/lower dentures, and having functional occlusal contacts [an optional measure states may collect in the older adult BSS protocol]) (2).

### Inclusion criteria and search strategy

We selected state reports in which 1) the average age of the study population was 65 years or older, 2) the oral health status was clinically assessed using BSS protocol, and 3) examinations were conducted after 2011. State surveys used in the analysis for aim 2 also had to be designed to obtain a statewide estimate of LTC residents.

We searched for states that had published findings (eg, report, presentation, journal article) from an older adult BSS. A list of these states was obtained from the ASTDD website (current as of 2019) and a report by Oral Health America (OHA; current as of 2016) (8). For each state, we searched for the most recent available report. If available, BSS reports were downloaded from the ASTDD website. For the remaining states, we searched the following: 1) Google (search strategy was [state name] older adults “BSS” or “basic screening survey”); 2) websites of state oral health programs; and 3) *Journal of Dental Hygiene* (search strategy was [state name] “older adults”). We searched the *Journal of Dental Hygiene* separately because most peer-reviewed BSS reports that we located were published in this journal. Finally, to confirm that we had not missed any state BSS reports, we searched PubMed, ScienceDirect, and Scopus using the following search terms: (“Basic Screening Survey” or “BSS”) AND “oral health” AND (“older adult*” OR “elderly” OR “geriatric” OR “senior*”). We also contacted the ASTDD Healthy Aging Committee and state oral health departments (for states funded by the Centers for Disease Control and Prevention Division of Oral Health) (9) to seek help in locating reports.

### Data extraction

We developed a form to extract information on survey population characteristics (eg, LTC or community dwelling, socio-demographic characteristics) and items described previously to assess heterogeneity in reporting of study conduct and oral health status. To assess the utility of this form and ensure similar abstraction standards, we independently abstracted 2 BSS reports, compared content, and revised the abstraction form as needed. Once the form was finalized, one author (M.L.) reviewed selected reports to ensure they met inclusion criteria.

Included reports were reviewed and extracted by 2 researchers to ensure accuracy. M.L. extracted data from all reports, and S.G. and E.H. served as second reviewers. Reviewers compared, discussed, and reached consensus on their extraction findings. The statistician reviewer (E.H.) extracted the information on survey conduct from all reports, and M.L. and S.G. served as second reviewers.

### Data analysis

For Aim 1, we calculated the number of states reporting each data element and qualitatively assessed heterogeneity across states. Indicators were determined to be “fully reported” if a state reported all recommended components of the BSS protocol, “partially reported” if a state reported some of the recommended components only, or “did not report” if the reporting did not align with the BSS protocol. For Aim 2, we calculated the median, mean, and ranges of the 3 indicators of preventable oral health conditions and the 4 indicators of dentition across states with comparable data (ie, states that reported data with the same numerator and denominator, consistent with the BSS protocol).

## Results

### Selected BSS reports

**Figure 1:**
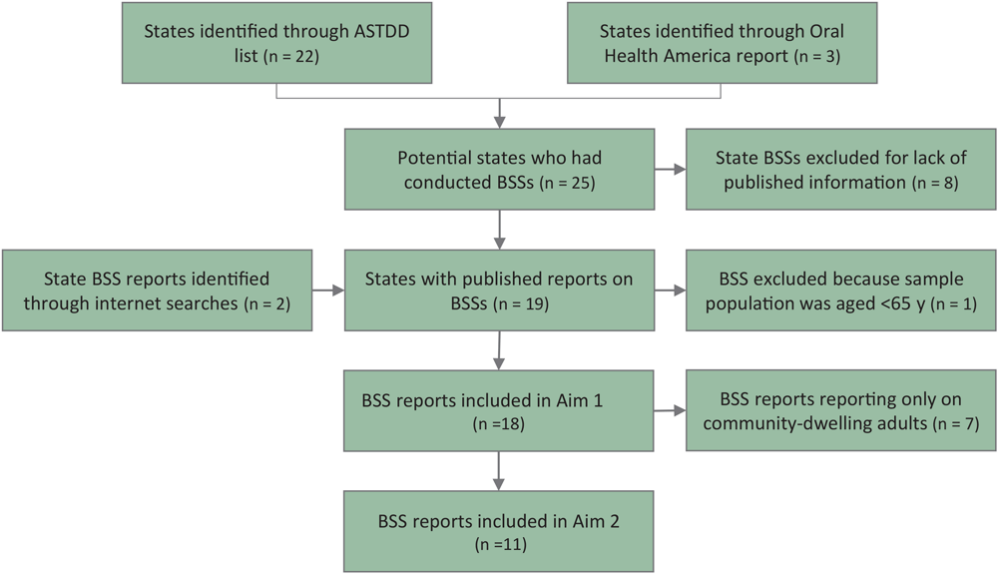
Process for identifying reports from the Basic Screening Survey (BSS) for older adults that met inclusion criteria. ASTDD, Association of State and Territorial Dental Directors.

We identified 22 states from ASTDD (Alaska, Arkansas, California, Connecticut, Florida, Georgia, Iowa, Kansas, Maryland, Michigan, Minnesota, Nebraska, New Hampshire, North Carolina, North Dakota, Oregon, Rhode Island, Vermont, Virginia, Washington, West Virginia, and Wisconsin) and 3 additional states from OHA (Delaware, Indiana, Kentucky) that had conducted an older adult BSS (Figure 1) after 2011. From these 25 states we located reports for 17 (Arkansas, California, Connecticut, Florida, Georgia, Iowa, Kansas, Maryland, Michigan, Minnesota, New Hampshire, North Carolina, North Dakota, Vermont, Washington, West Virginia, and Wisconsin). We located 2 additional reports (Indian Health Service [IHS], Texas) from our internet searches. Although the IHS report highlighted important information, we excluded it because the average age of the population was younger than 65 years. Searching biomedical databases and reaching out to states yielded no additional BSSs. Our final sample consisted of 18 states, of which 11 had statewide data for older adults living in LTC (Arkansas, California, Connecticut, Georgia, Kansas, Maryland, Minnesota, North Carolina, North Dakota, Vermont, Wisconsin) (10–27).

The states that made up the samples were geographically diverse. Of 18 states for Aim 1, 8,564 institutionalized adults from at least 341 LTC sites and 7,198 community-dwelling adults from at least 347 community locations (eg, congregate meal sites, senior centers; Georgia and West Virginia site numbers unclear) were sampled (a total of 15,762 older adults). Of 11 states for Aim 2, 8,564 institutionalized adults were sampled from at least 341 LTC sites.

### Aim 1a: heterogeneity in reporting study conduct

Fourteen of the 18 states indicated completing examiner training and/or calibration. No state reported any measures or indicators of screener agreement or reliability (Table 1). Seven of 18 states specified using a consultant for technical assistance. BSS reports from an additional 3 states had authorship showing a statistician, an epidemiologist, or an ASTDD consultant, but their roles were not specifically reported.

**Table 1:** Heterogeneity in Reporting How Older Adult Basic Screening Surveys Were Conducted in Selected US States, 2011–2019

State	Examiners		Use of Consultant	Site Selection Methods	No. Sites^a^ (Response Rate, %)		No. Participants^a^ (Response Rate, %)		Adjustment of Data^b^	95% CIs Provided
	No.	Reported Training								
					LTC	CD	LTC	CD		
Arkansas	10	Yes	NR	Probability	118 (88)	85 (92)	1,646 (82)	1,077 (78)	Yes	Yes
California	NR	Yes	Yes	LTC, probability; CD, convenience	36 (NR)	51 (NR)	1,193 (NR)	1,179 (NR)	NR	No
Connecticut	NR	Yes	NR^c^	LTC, probability; CD, convenience	8 (40)	16 (NR)	419 (NR)	426 (NR)	NR	No
Florida	17	Yes	NR	Probability	—	35 (100)	—	668 (21)	Yes	Yes
Georgia	NR	Yes	NR	Convenience	NR	NR	362 (NR)	201 (NR)	NR	No
Iowa	23	Yes	Yes	Probability	—	46 (NR)	—	736 (55)	Yes	No
Kansas	NR	NR	Yes	NR	20 (NR)	—	540 (NR)	—	NR	No
Maryland	4	Yes	NR	NR	40 (NR)	39 (NR)	488 (NR)	506 (NR)	NR	No
Michigan	3^d^	Yes	Yes	Convenience	—	R1: 8 (NR); R2: 21 (NR)	—	R1: 350 (NR); R2: 397 (NR)	Yes	No
Minnesota	NR	Yes	Yes	Probability	31 (100)	—	944 (NR)	—	Yes	Yes
New Hampshire	10	Yes	NR^c^	Probability	—	25 (100)	—	610 (NR)	Yes	Yes
North Carolina	NR	Yes	NR	Probability	40 (100)	—	854 (NR)	—	Yes	Yes
North Dakota	NR	NR	NR	NR	13 (NR)	—	579 (NR)	—	NR	No
Texas	2	Yes	NR	NR	—	6 (NR)	—	78 (NR)	NR	No
Vermont	2	NR	Yes	Probability	8 (100)	—	342 (NR)	—	Yes	No
Washington	NR	NR	NR^c^	Convenience	—	15 (NR)	—	570 (NR)	Yes	No
West Virginia	NR	Yes	NR	Unclear^e^	NR	NR	—	400 (NR)	NR	No
Wisconsin	NR	Yes	Yes	Probability	27 (NR)	—	1,197 (NR)	—	Yes	Yes

Abbreviations: — , not applicable; ASTDD, Association of State and Territorial Dental Directors; CD, community-dwelling adults; LTC, long-term care or skilled nursing facility; NR, not reported; R1, region 1; R2, region 2.

a Only 2 state reports (New Hampshire and North Carolina) included text suggesting a predetermined minimum sample size. Community-dwelling adults were those at congregate meal sites or senior sites.

b Reporting of weighted data analysis, adjusting for sampling scheme, varying probability of selection, and/or nonresponse.

c Authorship specifically lists a statistician, an epidemiologist, or an ASTDD consultant, but role is not specified.

d The same 2 registered dental hygienists and 1 assistant staffed all but 3 sites. These 3 sites required additional hygienists to be calibrated and used.

e Reported “a random convenience sampling strata of the meal sites was used to assure a representative sample of the entire state.”

Thirteen states reported on sampling methods for site selection. Ten of them reported selecting sites by using probability sampling methods. Among them, 2 also used convenience sampling for a second population group. Three more states reported using convenience sampling for site selection. Five states reported little or no information about site selection.

Regarding participant selection methods, 4 states did not report any information. Four other states provided minimal information such as “adults were offered screening,” “residents volunteered,” or implication of a convenience sample. Seven states indicated efforts to screen all eligible and consenting adults at the selected facilities. Three more states indicated an effort to screen all eligible and consenting adults but that access to participants was driven by facility staff.

Only 1 state specified a sample size based on study design and preferred precision, although this precision was not clarified. One state included a description that implied a predetermined sample size, but no specific information on methods was given. The remaining 16 states made no mention of sample size considerations. However, other study design efforts and technical assistance in many states imply that there was likely some consideration of sample size needs but reporting details of this survey stage were lacking.

For survey response rate, 16 states provided the number of sites included in the survey. Of them, 7 states reported the site response rate or provided enough information to ascertain it. All 18 states provided the number of participants surveyed. Three reported the participant response rate or provided enough information to ascertain it.

Ten states reported using weighted data analysis to increase representativeness of the study population. Of 8 remaining states, none specifically stated that findings were unadjusted. Six states provided 95% confidence interval values for reported measures.

### Aim 1b: heterogeneity in reporting oral health outcomes

All states reported the prevalence of untreated decay. One state reported untreated caries lesions and root caries lesions separately, which is not in alignment with the ASTDD protocol. One state reported untreated decay by sex and ethnicity but not as one composite value.

Sixteen of the 18 states completely reported the prevalence of need for periodontal care. One state reported the indicator by sex and ethnicity but not as one composite value. 

Seventeen states reported need for dental care. Thirteen reported 2 categories (early and urgent), and 4 reported 1 category (any need for dental care; partially reported). Two states did not follow the BSS protocol to report this indicator for dentate adults and instead reported for all survey participants.

Although edentulism (total tooth loss) is not part of the BSS protocol, 17 states reported it. 

Four states fully reported the number of natural teeth by reporting the mean number of teeth in the maxillary and mandibular arches. Three states partially reported the mean number of natural teeth for the mouth, not stratifying by arch. An additional 3 states partially reported on the presence of natural teeth using cutoff numbers: the first state indicated the percentage of respondents who were missing 1 to 6 teeth or more than 6 teeth, the second state indicated the percentage of respondents with 0 to 5 missing teeth or more than 5 missing teeth, and the last state indicated the percentage of respondents with “impaired dental function,” defined as having fewer than 20 teeth.

Fourteen of the 18 states reported at least 1 value for denture ownership or use. Compared with the other indicators, reporting for the denture ownership or use was the most common variation. Nine states fully reported the percentage of survey respondents who had upper dentures. One state partially reported denture ownership by stating the percentage of participants who owned any denture (not specifically upper or lower). Three states did not use the protocol-specified denominator (all participants): 2 states calculated the ownership among edentulous adults while another state calculated the ownership among occlusion-deficient residents.

For denture use, 5 states fully reported the prevalence of those with removable upper dentures who wore them while eating and one state slightly deviated from the BSS question by reporting the percentage of older adults who removed their dentures before eating. One state partially reported denture use by stating only the prevalence of those who “use” upper dentures. A similar reporting pattern was also found in reporting on ownership and use of lower dentures.

Four states fully reported the prevalence of functional occlusal contacts by reporting those who had functional occlusal contacts on both sides and on 1 side only. Three states partially reported this indicator by reporting those who had functional occlusal contacts on 1 side only and no sides. One state reported only those who had no functional occlusal contacts. Several states presented the data in multiple ways, including among the dentate, edentulous, and all respondents. One state indicated that it had collected the data but did not report it. 

### Aim 2: synthesis of outcomes for LTC

#### Preventable conditions

**Figure 2:**
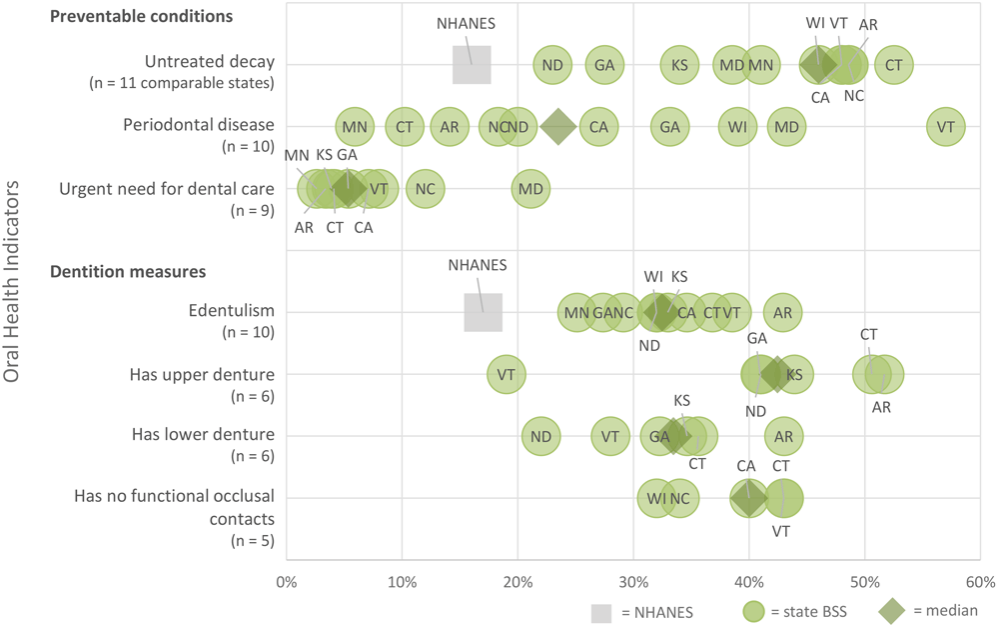
Prevalence of oral health indicators among older adults in long-term care (LTC) facilities as reported in state basic screening surveys. Abbreviations: BSS, basic screening survey; NA, not applicable; NHANES, National Health and Nutrition Examination Survey; NR, not reported.

The reported prevalence of untreated decay in LTC facility residents (n = 11 states with comparable data) ranged from 23% to 53% (Figure 2), with a median value of 46% and a mean value of 41%.

**Table:** 

State	Untreated Decay, %	Periodontal Disease, %	Urgent Need for Dental Care, %	Edentulism, %	Has Upper Denture, %	Has Lower Denture, %	Has No Functional Occlusal Contacts, %
Arkansas	49	14	3	43	52	43	NR
California	48	27	7	35	NR	NR	40
Connecticut	53	10	4	37	51	36	43
Georgia	28	33	5	27	4	32	NR
Kansas	34	NR	4	33	44	35	NR
Maryland	39	43	21	NR	NR	NR	NR
Minnesota	41	6	3	25	NR	NR	NR
North Carolina	49	18	12	29	NR	NR	34
North Dakota	23	20	NR	32	41	22	NR
Vermont	48	57	8	39	19	28	43
Wisconsin	46	39	NR	32	NR	NR	32
Median	46	24	5	32	42	33	40
NHANES	16	NA	NA	17	NA	NA	NA

The reported prevalence of a need for periodontal care among all surveys of LTC facility residents (n = 10 states with comparable data) ranged from 6% to 57% (median, 24%; mean, 27%). The reported prevalence of an urgent need for dental care among all surveys of LTC facility residents (n = 9 states with comparable data) ranged from 3% to 21% (median, 5%; mean, 8%).

#### Dentition measures

The reported prevalence of edentulism among all surveys of LTC facility residents (n = 10 states with comparable data) ranged from 25% to 43% (median, 32%; mean, 33%). Data on the number of natural teeth were not reported consistently across selected states; no comparison could be made (Table 2).

**Table 2:** Heterogeneity in Reporting Oral Health Outcomes From Older Adult Basic Screening Surveys, Selected US States, 2011–2019

State	Preventable Conditions			Dentition				
	Untreated Decay	Periodontal Care	Need for Dental Care	Edentulism	Number of Natural Teeth	Denture Ownership	Denture Use	Functional Occlusal Contacts
Arkansas	⬤	⬤	⬤	⬤	◑	⬤	⬤	◯
California	⬤	⬤	⬤	⬤	◑	◍	◯	◑
Connecticut	⬤	⬤	⬤	⬤	⬤	⬤	⬤	⬤
Florida	⬤	⬤	⬤	⬤	◑	◯	◑◍	◯
Georgia	⬤	⬤	⬤	⬤	◯	⬤	◯	◯
Iowa	⬤	⬤	⬤	⬤	◯	⬤	⬤	◯
Kansas	⬤	◯	⬤	⬤	◯	⬤	◯	◯
Maryland	⬤	⬤	⬤	⬤	◯	◯	◯	◯
Michigan	⬤◉	⬤◉	◑◍	⬤	◯	⬤◉	⬤◉	⬤◉
Minnesota	⬤	⬤	⬤	⬤	◑	◑	◯	⬤
New Hampshire	⬤	⬤	⬤	⬤	⬤	⬤	⬤	◑
North Carolina	⬤	⬤	⬤	⬤	◑	◍	◯	◑
North Dakota	⬤	⬤	◑◍	⬤	◯	⬤	◯	◯
Texas	◍◉	⬤◉	◯	◯	⬤◉	◯	◯	◯
Vermont	⬤	⬤	⬤	⬤	⬤	⬤	⬤	◑
Washington	⬤◉	◉	◑	⬤	◯	◯	◯	◯
West Virginia	⬤	◯	⬤	⬤	◯	◯	◯	◯
Wisconsin	⬤	⬤	◑	⬤	◑	◍	◯	⬤

Abbreviations: ⬤, fully reported based on ASTDD protocol; ◉, broken down by demographics; ◑, partially reported; ◯, not reported; ◍, reporting does not align with ASTDD protocol; ASTDD, Association of State and Territorial Dental Directors.

The reported prevalence of upper dentures among all surveys of LTC facility residents (n = 6 states with comparable data) ranged from 19% to 52% (median, 42%; mean, 41%). Data on the prevalence of use of upper dentures during eating was not reported consistently across states (Table 2).

The reported prevalence of lower dentures among all surveys of LTC facility residents (n = 6 states with comparable data) ranged from 22% to 43% (median, 33%; mean, 33%). Data on the prevalence of use of lower dentures during eating was not reported consistently across states (Table 2).

The reported prevalence of a lack of functional occlusal contacts among all surveys of LTC facility residents (n = 5 states with comparable data) ranged from 32% to 43% (median, 40%; mean, 38%).

## Discussion

The BSS fulfills states’ need for oral health surveillance data for older adults. Its use among states has steadily increased since its introduction in the early 2000s (5). At present, at least 26 states have conducted 1 or more BSSs among older adults. Given its wide use and acceptance, developing reporting guidelines and providing detailed instructions on outcome calculations is important.

Our analysis showed variation in states’ reporting of older adult BSS study conduct and outcomes. Variation was especially notable among states’ reporting of sampling, screening, and statistical methods. Although most states reported basic sampling information such as site selection method, number of sites, and number of participants, many did not provide more specific, but important, sampling details such as participant selection methods or response rate information. Many states also did not report whether data were adjusted or report measures of error (such as confidence intervals) for reported outcomes. The older adult BSS guidelines for study conduct are less prescriptive than those that describe which outcomes to collect. The BSS instructions note that, although statistical design and methods are important considerations, practical resource limitations exist that may have a greater impact on survey conduct than statistical rigor (5). The practicality of striking this balance in a real-life setting highlights the importance of full reporting of methods so that the audience may understand the level of precision, have confidence in the findings, and know the comparability to other studies. It is also likely that many of the indicators of the quality of study conduct, although unreported, may have been met. Seven of the 18 states consulted with an epidemiologist or statistician, and 3 additional states mentioned the contribution from an epidemiologist/statistician as a coauthor of their BSS reports.

We also found variation in reporting some outcomes, especially those related to tooth loss and denture use. Tooth loss can affect the ability to eat healthy foods (28) and likely has the strongest influence on quality of life. It has the highest disability weight (loss in quality of life due to living with condition for 1 year) of the 3 oral conditions monitored by the Global Burden of Disease, 0.073, with 0.012 for untreated caries and 0.0079 for severe periodontitis (28). Differences in included outcomes may have been due to specific state needs. Comparability of BSS findings across states, however, would be enhanced if all states reported some minimal set of standardized measures for tooth loss and denture use in addition to measures tailored for specific state needs.

We also found that different formulae were used in the calculation of some reported outcome measures, including using different denominators (ie, calculating prevalence among edentulous survey participants instead of all participants). Detailed instruction on measure calculations, similar to that provided by the National Oral Health Surveillance System (29), could further improve comparability.

ASTDD took the lead in developing a standardized, resource-efficient protocol to assess the oral health status of older adults, without which we would have no data on the oral health status of LTC residents in the United States, nor state-level estimates for older adults overall. Information regarding LTC residents’ oral health is scarce. Although the Centers for Medicare and Medicaid Services requires all certified nursing homes in the United States to perform a comprehensive assessment of each resident's functional capabilities and health, the resulting Long-Term Care Minimum Data Set includes only 1 dichotomous variable on overall oral health status (30).

Although findings of older adult BSS reports have been combined to examine prevalence of edentulism and untreated caries among long-term care residents (3), ours is the first study to systematically review and synthesize findings of BSS reports. In addition, prior to our study, no studies had synthesized periodontitis, urgent unmet treatment needs, and measures of tooth retention other than edentulism. Our review suggests that older adults in LTC facilities have worse oral health compared with their community-dwelling counterparts. Outcomes for LTC adults from this study that appeared to be higher than reported estimates from 2011–2016 National Health and Nutrition Examination Survey data for community dwelling older adults included 1) untreated caries (median prevalence across 11 states, 46% vs 16%) and 2) edentulism (median prevalence across 10 states, 32% vs 17%) (1).

This study was subject to at least 2 limitations. First, some BSS reports may have been excluded; information on the ASTDD website indicated that BSSs were conducted in certain states for which we could not locate reports. Second, the population in LTC from selected BSS reports may not be representative of the state LTC population aged 65 years or older; for some state reports, we included residents younger than 65, and although LTC facilities were selected randomly, residents in each facility were typically a convenience sample.

Although obtaining state representative estimates of oral health through clinical assessment is resource intensive, many US states have deemed that the information gained from these assessments outweighs the costs. Our findings suggest that more standardized reporting of BSS conduct and outcomes could improve users’ ability to assess the validity and reliability of information and to compare survey findings among participating states.

## Appendix Basic Screening Survey Indicator Definitions^a^

**Table:**

Indicator	Definition	Denominator
Untreated decay	Presence of readily observable breakdown of the enamel or cementum.	Dentate participants
Need for periodontal care	If a participant needs to have their teeth cleaned before their next regularly scheduled dental appointment or more advanced periodontal treatment.	Dentate participants
Treatment urgency	Urgent need for dental care: a participant needs dental care within the next week because of signs or symptoms that include pain, infection, or swelling. Early dental care needed: a participant needs to see a dentist because of untreated decay, but they do not have pain or infection.	All participants
Number of natural teeth	Number of natural teeth present in the upper arch and the number of natural teeth present in the lower arch, including third molars, retained primary teeth and root fragments.	All participants^b^
Dentures and denture use	Participants were asked “Do you have a removable upper denture?” If they answered yes, they were asked “Do you usually wear your denture when you eat?” The same questions were asked for lower dentures.	Denture ownership: all participants; denture use: participants who own that denture
Functional posterior occlusal contacts	Whether or not the participant has opposing pairs of natural or non-natural posterior teeth. This is assessed with removable dentures in place, when appropriate.	All participants

^a^ Definitions from Association of State and Territorial Dental Directors Older Adult BSS Protocol (5).

^b^ Participants who have no natural teeth were identified as edentulous.
